# Prosthetic joint replacement for femoral bone metastases and pathological fracture: A retrospective case series study of cases within the past 10 years

**DOI:** 10.1097/MD.0000000000046927

**Published:** 2025-12-26

**Authors:** Ichiro Itonaga, Yuta Kubota, Masanori Kawano, Kazuhiro Tanaka, Nobuhiro Kaku

**Affiliations:** a Department of Orthopaedic Surgery, Faculty of Medicine, Oita University, Yufu-city, Oita, Japan; b Department of Advanced Medical Sciences, Faculty of Medicine, Oita University, Yufu-city, Oita, Japan.

**Keywords:** bone metastasis, pathological fracture, prosthetic joint replacement

## Abstract

This study investigated the postoperative outcomes of patients with pathological fractures who had undergone tumor resection and reconstruction using tumor endoprostheses for femoral bone metastases over the past 10 years. We conducted a retrospective case series of patients who underwent tumor resection and endoprosthetic reconstruction for femoral metastases and pathological fractures between January 2016 and December 2024. The minimum follow-up period was 6 months or until death. Postoperative survival was the primary outcome. Statistical analyses were performed using the IBM SPSS Statistics version 25. Kaplan–Meier survival curves were generated, and the chi-square and Mann–Whitney *U* tests were used for comparisons. Eighteen patients were retrospectively analyzed. The cohort included 7 men and 11 women, with a median age of 68.5 years (range, 38–88 years). Primary malignancies included breast cancer (n = 8), renal cell carcinoma (n = 4), prostate cancer (n = 2), and one case each of non-small cell lung cancer, follicular thyroid carcinoma, gastric cancer, and hepatocellular carcinoma. At the final follow-up, 7 patients were alive and 11 had died. Fifteen patients (83.3%) survived for more than 6 months after surgery. The estimated median postoperative survival time was 18 months. Among the 16 patients who received postoperative chemotherapy, the estimated median survival was 41 months. Postoperative chemotherapy was significantly associated with survival of ≥ 6 months (*P* < .05). Reconstruction using tumor endoprostheses appears to be a reliable and effective surgical option for patients with femoral bone metastases and pathological fractures, particularly when postoperative chemotherapy can be administered. However, these findings should be interpreted with caution given the retrospective design and limited sample size.

## 1. Introduction

Recent advances in the treatment of malignant tumors have significantly prolonged patient survival in various cancers. As survival outcomes improve, oncologic care has increasingly focused not only on life extension but also on enhancing patients’ quality of life.

Bone metastases to the lower extremities, particularly those causing pathological fractures, are a major cause of morbidity in cancer patients. These fractures often cause severe pain and substantial functional impairment, especially mobility, which greatly diminishes the quality of life. Once a pathological fracture occurs, the prognosis depends on multiple factors, including the type of primary tumor, overall health status, and available treatments.^[1–4]^ Therefore, surgical intervention should be carefully considered, considering the patient’s overall condition and anticipated prognosis.

Among surgical options, resection of metastatic bone lesions followed by reconstruction using tumor endoprostheses has emerged as an effective and durable strategy. This approach is particularly beneficial when long-term survival is expected because it provides stable limb function and sustained pain relief.^[5,6]^ However, postoperative survival outcomes in recent years remain incompletely understood.

Evaluating the outcomes of such surgical interventions is critical, given the increasing number of cancer patients with prolonged survival. The aim of this study was to investigate the postoperative survival of patients who underwent tumor resection and reconstruction with tumor endoprostheses for femoral metastases and pathological fractures over the past decade.

## 2. Methods

This retrospective case series included all patients who underwent tumor resection and reconstruction using tumor endoprostheses for femoral metastases or pathological fractures at our institution between January 2016 and December 2024. Surgical procedures were performed in consultation with the primary oncologist and were generally indicated for patients with an expected survival of at least 6 months. The minimum follow-up period was 6 months or until death. The primary outcome was postoperative survival.

Statistical analyses were performed using IBM SPSS Statistics version 25 (IBM Corp., Armonk). Kaplan–Meier survival curves were generated for all patients and for those who received postoperative chemotherapy. To identify factors associated with postoperative survival of 6 months or longer, chi-square tests were conducted for the following variables: receipt of postoperative chemotherapy, use of preoperative bone-modifying agents, number of bone metastases (solitary or multiple), and presence of visceral metastases. The intervals from cancer diagnosis to surgery, operative time, and intraoperative blood loss were analyzed using the Mann–Whitney *U* test.

## 3. Results

All surgeries were performed by 3 orthopedic surgeons at our institution. The study population comprised 18 patients, including 7 men and 11 women, with ages ranging from 38 to 88 years (median, 68.5 years) (Table 1). Primary malignancies included breast cancer (n = 8), renal cell carcinoma (n = 4), prostate cancer (n = 2), and one case each of non-small cell lung cancer, follicular thyroid carcinoma, gastric cancer, and hepatocellular carcinoma (Table 1). Pathological fractures were located in the proximal femur in 17 cases and in the distal femur in one case (Table 1).

**Table 1:** Characters of patients.

Gender	Age	Primary malignancies	Site of pathological fracture
Male: 7 Female: 11	38–88 (median:68.5)	Breast cancer: 8 Renal cell carcinoma: 4 Prostate cancer: 2 Non-small cell lung cancer: 1 Follicular thyroid carcinoma: 1 Gastric cancer: 1 Hepatocellular carcinoma: 1	Proximal femur: 17 Distal femur: 1

**Figure 1.**
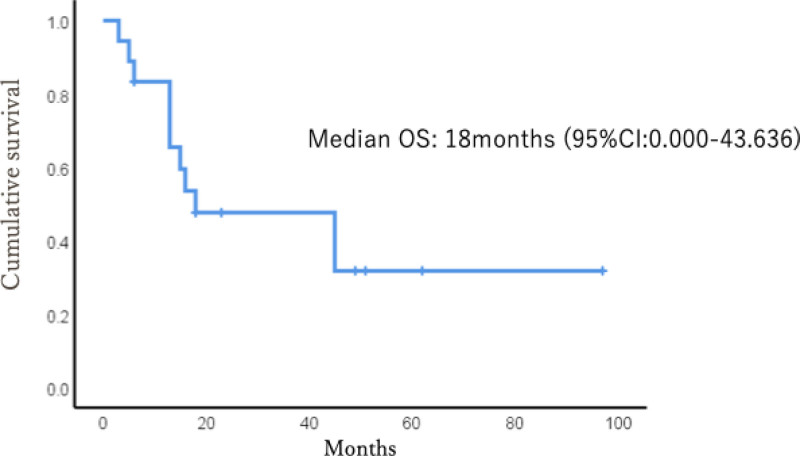
Kaplan–Meier curve for median OS. OS = overall survival.

At the final follow-up, 7 patients were alive and 11 died. Fifteen patients (83.3%) survived longer than 6 months postoperatively, including 2 who survived for over 5 years. The estimated median postoperative survival time was 18 months (Fig. 1). Of the 3 patients who died within 6 months after surgery, 2 had non-small cell lung cancer and hepatocellular carcinoma, respectively, both experiencing postoperative declines in performance status that precluded chemotherapy.

**Figure 2.**
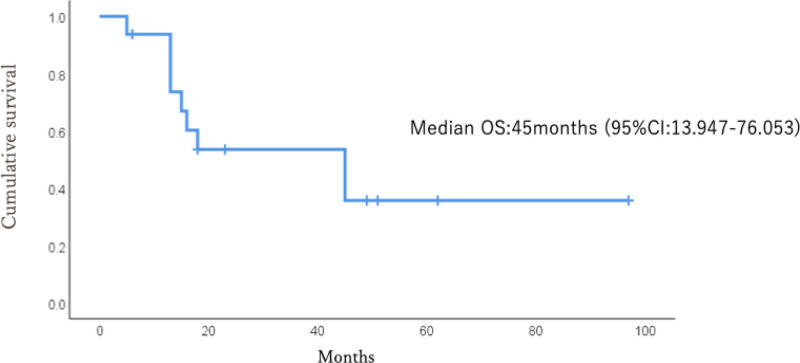
Kaplan–Meier curve of cases that underwent postoperative chemotherapy for median OS. OS = overall survival.

The estimated median survival of the 16 patients who received postoperative chemotherapy was 45 months (Fig. 2). Postoperative chemotherapy was significantly associated with survival beyond 6 months (chi-squared test, *P* = .0189) (Table 2). No prosthesis failure due to bacterial infection or local postoperative recurrence was observed.

**Table 2:** Clinical and surgical characteristics and statistical analysis of factors associated with postoperative survival ≥ 6 months.

Factor	Test	*P* value	Significant (*P* < .05)
Postoperative chemotherapy (cases with/ without) Received: 16 Not received: 2	Chi-square test	.019	Yes
Use of bone-modifying agents (cases) Received: 4	Chi-square test	1.000	No
Bone metastasis pattern (solitary/ multiple) Solitary: 3 Multiple: 15	Chi-square test	1.000	No
Visceral metastasis (sites/ cases) Present: 8 Lung: 4, Liver: 1, Retroperitoneum: 1, Skin: 1, Subcutaneous: 1	Chi-square test	1.000	No
Duration from cancer diagnosis to surgery (mo) Range: 0–156 (median: 8)	Mann–Whitney *U* test	.765	No
Operation time (min) Range: 127–278 (median: 172)	Mann–Whitney *U* test	.097	No
Intraoperative blood loss (mL) Range: 150–1500 (median: 460)	Mann–Whitney *U* test	.953	No

In some cases, the interval from cancer diagnosis to pathological fracture was up to 13 years. Bone-modifying agents were administered prior to fracture in 4 patients, while pathological fracture was the initial manifestation of malignancy. At surgery, bone metastases were solitary in 3 cases and multiple in 15 cases. Extraosseous metastases included lung metastases in 4 cases and other sites in 4 cases; 10 patients had no extraosseous metastases (Table 2).

Surgical duration ranged from 2 hours 7 minutes to 4 hours 38 minutes (median, 2 hours 52 minutes), and intraoperative blood loss ranged from 150 to 1500 mL (median, 460 mL). None of these factors were significantly associated with postoperative survival (Table 2).

## 4. Discussion

The development and clinical use of novel molecular targeted therapies and immune checkpoint inhibitors have improved cancer treatment outcomes. The median overall survival for stage IV breast cancer exceeds 3 years,^[7]^ with approximately 29 months for prostate cancer,^[8]^ 16.8 months for renal cell carcinoma,^[2]^ and 14.6 months for gastric cancer,^[3]^ Additionally, a 10-year survival rate of 42% has been reported for stage IV follicular thyroid carcinoma.^[4]^ Several studies have shown prolonged survival with these new agents compared with conventional treatments, and further improvements are anticipated.^[9–13]^

As survival in advanced cancer has improved, the risk of bone metastases and pathological fractures has increased. The femur is the most common site of metastasis in the long bones.^[14]^ Surgical intervention should be considered when patients experience severe pain or weight-bearing difficulties. For patients with an expected long-term survival, surgical options that provide durable local control and stable long-term outcomes are necessary. Tumor resection followed by reconstruction with tumor endoprostheses is one such treatment option. In this study, one patient underwent tumor endoprosthetic replacement 8 years and 5 months after cancer diagnosis and remained alive 8 years postoperatively.

The prognosis of cancer patients with bone metastases varies widely, making accurate survival prediction challenging. The estimated median postoperative survival time in this study was 18 months. Among the patients who received postoperative chemotherapy, the median survival time was 41 months, and chemotherapy was significantly associated with prolonged survival. These findings suggest that the ability to receive postoperative chemotherapy and availability of effective systemic therapies are important determinants of long-term survival. From a clinical perspective, these results underscore the importance of appropriate patient selection when considering tumor endoprosthetic reconstruction for femoral bone metastases. Furthermore, in patients who survived for extended periods after postoperative chemotherapy, tumor resection followed by reconstruction using endoprostheses proved to be a stable and effective surgical option.

However, some patients may be unable to receive postoperative chemotherapy due to clinical deterioration. In this study, 2 patients, one with non-small cell lung cancer and the other with hepatocellular carcinoma, did not receive chemotherapy and died 3 and 6 months post-surgery, respectively. David et al reported a median survival of approximately 2 months for untreated stage IV non-small cell lung cancer.^[15]^ Similarly, Kakizaki et al^[16]^ reported about 2 months for untreated stage IV hepatocellular carcinoma. Even when postoperative chemotherapy is planned, factors may prevent treatment, potentially resulting in a poorer prognosis than that initially expected.

The limitations of this study include its retrospective design and single-institution nature, as well as the small sample size, which limits the statistical reliability of the analysis. In addition, the predominance of breast cancer cases (44.4%) may have influenced the overall outcomes.

## 5. Conclusion

We examined the postoperative outcomes of patients who underwent tumor resection and reconstruction using tumor endoprostheses for femoral and pathological fractures. Tumor endoprosthetic reconstruction is a valuable surgical option for patients with femoral bone metastases and pathological fractures, especially when postoperative chemotherapy is feasible, as it may contribute to extended survival. However, these findings should be interpreted with caution due to the retrospective design and the limited sample size.

## Author contributions

**Conceptualization:** Ichiro Itonaga.

**Data curation:** Yuta Kubota, Masanori Kawano.

**Investigation:** Ichiro Itonaga.

**Supervision:** Kazuhiro Tanaka, Nobuhiro Kaku.

**Writing – original draft:** Ichiro Itonaga.
